# New reverse genetics and transfection methods to rescue arboviruses in mosquito cells

**DOI:** 10.1038/s41598-017-14522-6

**Published:** 2017-10-25

**Authors:** Thérèse Atieh, Antoine Nougairède, Raphaëlle Klitting, Fabien Aubry, Anna-Bella Failloux, Xavier de Lamballerie, Stéphane Priet

**Affiliations:** 1 UMR “Émergence des Pathologies Virales” (EPV: Aix-Marseille Univ – IRD 190 – Inserm 1207 – EHESP – IHU Méditerranée Infection), Marseille, France; 2 Arboviruses and Insect Vectors Unit, Department of Virology, Pasteur Institute, Paris, France

## Abstract

Reverse genetics is a critical tool to decrypt the biological properties of arboviruses. However, whilst reverse genetics methods have been usually applied to vertebrate cells, their use in insect cells remains uncommon due to the conjunction of laborious molecular biology techniques and of specific difficulties surrounding the transfection of such cells. To leverage reverse genetics studies in both vertebrate and mosquito cells, we designed an improved DNA transfection protocol for insect cells and then demonstrated that the simple and flexible ISA (Infectious Subgenomic Amplicons) reverse-genetics method can be efficiently applied to both mammalian and mosquito cells to generate in days recombinant infectious positive-stranded RNA viruses belonging to genera *Flavivirus* (Japanese encephalitis, Yellow fever, West Nile and Zika viruses) and *Alphavirus* (Chikungunya virus). This method represents an effective option to potentially overcome technological issues related to the study of arboviruses.

## Introduction

Arboviruses (Arthropod-borne viruses) constitute a large group of viruses carried and spread by blood feeding arthropods, especially mosquitoes, ticks and sandflies. They can be transmitted to a variety of vertebrates and are responsible for significant morbidity and mortality amongst humans and farmed animals globally. Arboviral diseases in humans range from mild febrile illness to severe encephalitis or haemorrhagic fever^1^. Iterative outbreaks worldwide over the past decades have highlighted the emergence or re-emergence potential of arboviruses, which are thus considered to be significant public and animal health threats^1–3^. Most arboviruses of public health importance are single-stranded RNA viruses belonging to the families *Flavi*-, *Toga*-, or *Bunyaviridae*.

Research focusing on arboviruses knew dramatic progress thanks to the use of reverse genetics systems allowing the study of virus life cycles, understanding the effect of specific mutations on viral replication or pathogenesis, and designing new vaccine strategies^4,5^. However, these reverse genetics systems focused to date almost exclusively on mammalian cells. Since arboviruses life cycle involves replication in both invertebrate vectors and vertebrate hosts, a simple and universal reverse genetics method allowing producing recombinant arboviruses in both vertebrate and arthropod cells would obviously facilitate the study of arbovirus biological properties, of their genomic evolution or cell interactions and restrictions. This awaited knowledge could provide in the future the key elements needed to predict outbreaks and to find efficient therapy. Unfortunately, although most reverse genetics systems proved to be efficient to recover arboviruses from vertebrate cell lines (for reviews see^4,5^), very few studies have reported such systems for arthropod cells and especially for cells from *Aedes* mosquitoes, one of the most important arbovirus vectors globally^6^. Indeed, reverse genetics systems designed for positive-sense single-stranded RNA viruses in *Aedes* mosquito cells are typically based to date on the lipofection or electroporation of synthetic capped RNA transcripts generated by *in vitro* transcription from SP6-^7–9^ or T7 promoter-driven^10–16^ full-length viral cDNA constructs. A second system only used marginally and based on the direct transfection of a T7 promoter-driven infectious clone in an *Aedes* mosquito cell line stably expressing the T7 RNA polymerase was established to produce a minireplicon of the Bunyamwera negative-strand RNA virus^17^.

Nevertheless, these reverse genetics systems suffer from two main limitations. First, the construction of full-length viral cDNA clones remains difficult and time consuming. To circumvent this issue, we recently developed a novel bacterium-free method of reverse genetics called ISA (Infectious Subgenomic Amplicons)^18^. The ISA procedure does not require a cloning step, propagation of cDNA plasmid clone into bacteria or *in vitro* RNA transcription. The concept of ISA is based on the production by PCR of 3 to 6 overlapping linear non-infectious subgenomic DNA fragments that encompass the entire viral genome, each with 70–100 bp overlapping regions. The first and last fragments were flanked respectively in 5′and 3′ by the CMV promoter and the hepatitis delta ribozyme followed by the simian virus 40 polyadenylation signal. Amplicons are mixed and transfected directly into susceptible cells to enabled the recovery of infectious viruses thanks to yet unknown *in cellulo* recombination events. Giving the possibility to generate infectious single-stranded positive-sense RNA viruses in mammalian cells within days, this method was previously successfully applied to a wide range of viruses belonging to genera *Flavivirus*, *Enterovirus* and *Alphavirus*. However, whether the ISA method could be adapted to *Aedes* mosquito cells remained to be demonstrated. Second, previous studies using electroporation and/or transfection with regular lipids-based reagents of RNA in *Aedes* mosquito cells demonstrated a very low efficiency of RNA delivery (ranging from 0.2 to 10%)^14,15^. Therefore, new powerful method of nucleic acids delivery in *Aedes* mosquito cells is needed and especially to be applied to the ISA reverse genetics system.

Here, we describe an improved transfection procedure for *Aedes* mosquito cells. Combined with the ISA reverse genetics system, it allowed recovering arboviruses in mosquito cells within days, demonstrating that the ISA method is effective in such cells and laying the foundations for a simple reverse genetics method allowing the concurrent study of arboviruses in both vertebrate and invertebrate cells.

## Results

**Figure 1:**
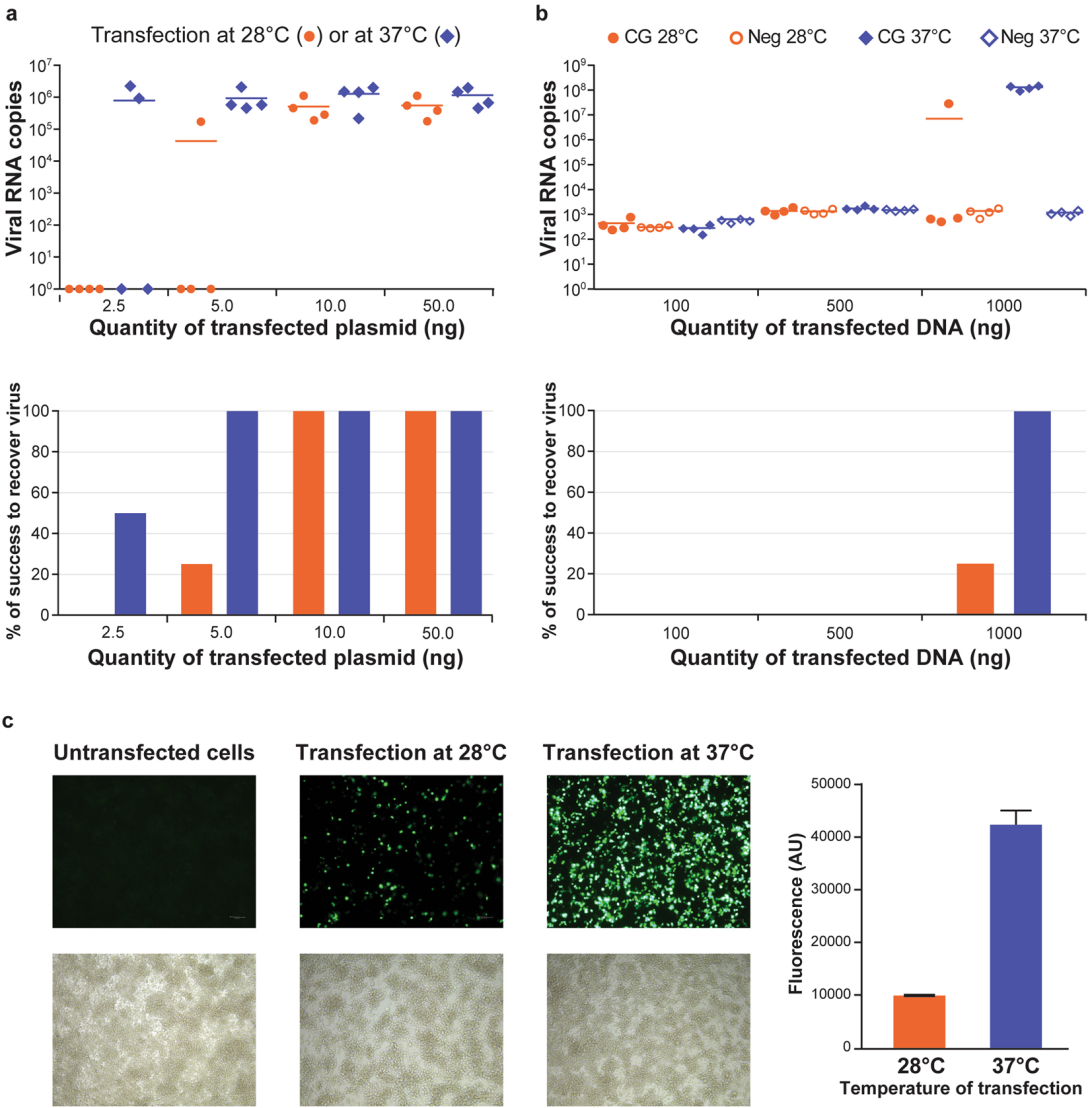
Effect of the temperature on the efficiency of virus rescue and on the efficacy of transfection in C6/36 cells. (**a**) West Nile Virus (WNV) infectious clone (2.5ng, 5ng, 10ng and 50ng) under the control of human cytomegalovirus promoter (pCMV) was transfected in C6/36 cells at 28 °C or 37 °C in quadruplicate. Viral RNA copies were quantified by RT-qPCR in cell supernatant at the first passage (upper panel). The % of success to recover a virus is reported in the lower panel. **(b**) ISA procedure was applied to WNV by transfecting 3 overlapping DNA amplicons (100ng, 500ng and 1000ng) covering the entire viral genome under the control of human cytomegalovirus promoter (pCMV) in C6/36 cells at 28 °C or 37 °C in quadruplicate. Viral RNA copies were quantified by RT-qPCR in cell supernatant at the first passage (upper panel). The % of success to recover a virus is reported in the lower panel. (**c**) Expression of eGFP in C6/36 cells measured 2 days after transfection of the eGFP gene under the control of the *D. melanogaster* actin 5C promoter at 28 and 37 °C (left panel). Bar graph representing the relative fluorescence intensity (right panel).

Preliminary experiments had suggested very limited efficacy of the ISA method in *Aedes* mosquito C6/36 cells (not shown). This initial observation could be explained either (i) by the ineffective transfection of amplicons, (ii) by the absence of the cellular mechanisms that allow in vertebrate cells the specific recombination of amplicons, the transcription of the recombined DNA in a full-length RNA genomic molecule and its export from the nucleus to the adequate cytoplasmic compartment where virus replication occurs, or (iii) by the poor efficacy of the CMV promoter used in our constructs in mosquito cells. We hypothesized that the transfection may constitute the major bottleneck because, on the one hand, limitation to reverse genetics of RNA viruses in mosquito cells had been observed using *in vitro* transcribed RNA derived from plasmidic infectious clones that do not require the abovementioned recombination mechanisms, and, on the other hand, the cellular mechanisms implicated are ancestral and therefore likely to be observed in both vertebrate and invertebrate cells. We further hypothesized that the fluidity of the cellular membrane may critically influence the efficacy of transfection and that the temperature of growth of insect cells (usually 28–32 °C) may unfavourably impact this factor and therefore limit the transfection capacity of insect cells. Accordingly, we designed a first experiment in which a previously validated^19,20^ bacterial DNA plasmid clone of West Nile Virus (WNV, a mosquito-borne flavivirus) would be transfected in C6/36 *Aedes albopictus* mosquito cells in parallel at 28 °C and 37 °C for 12 hours, followed by cell growth at 28 °C. The WNV infectious clone used (pWNV, strain Uganda 1937) harboured at 5′ terminus the human cytomegalovirus immediate early enhancer/promoter (pCMV) and at its 3′extremity a hepatitis delta virus ribozyme sequence followed by the simian virus 40 polyadenylation signal sequence (HDR/SV40pA). The transfection protocol was adapted from Atieh *et al*.^21^, using Lipofectamine 3000 (Invitrogen) in quadruplicate assays in 12.5 cm^2^ culture flasks at 28 °C or 37 °C with increasing quantities of pWNV (2.5ng, 5ng, 10ng and 50ng). After 12 h of transfection, cells were washed and incubated for 7 days at 28 °C. Cell supernatant was passaged once using fresh C6/36 cells and viral genomes were quantified in the clarified supernatant at day 5. As reported in Fig. 1a, the efficiency to rescue a virus was higher at 37 °C, allowing reaching 25% efficacy with 2.5ng of plasmid and 100% efficacy from 5ng of plasmid. In comparison, 10ng were required to ensure 100% efficacy at 28 °C and molecular viral loads in supernatants were systematically lower than those observed at 37 °C. As expected, cytopathic effects (CPE) were always observed in positive cell cultures, confirming the recovery of infectious viruses with infectious titers ranging between 4.7 and 5.9 log10 TCID_50_/mL (Supplementary Table S1). The overall success rate of the experiment was therefore dependent on several parameters, including the transfection efficacy as well as the replication and spread from cell to cell. However, since cells were incubated for 7 days at 28 °C after 12 h of transfection, we assumed that the replication and spread from cell to cell is similar in all conditions and that only a difference in transfection efficacy might explain a difference in the efficiency of virus rescue. This hypothesis was confirmed by the transfection of the eGFP gene under the control of the *D. melanogaster* actin 5C promoter, which was previously shown to allow a strong expression in *Aedes* cells^22,23^ (Fig. 1c).

The same experiment was then repeated using the ISA method (Fig. 1b), by transfecting increasing amounts (100ng, 500 ng and 1 µg) of an equimolar mixture of three overlapping sub-genomic amplicons produced from pWNV (and treated by DpnI enzyme to remove any residual template plasmidic DNA), as previously described^18^. Transfection of the same amounts of an equimolar mixture containing only the first and the second sub-genomic amplicons was used as a negative control. As shown in Fig. 1b, transfecting 1 µg of DNA was necessary to obtain a significant virus production. This dose ensured a 100% efficacy at 37 °C and only a 25% efficacy at 28 °C. Viral loads in supernatants were systematically lower than those observed at 37 °C. The immunofluorescent staining for viral antigens (Supplementary Figure S1), including at least the envelop glycoproteins and NS5 protein, showed that when the transfection is performed at 37 °C the virus rescue efficiency at the level of the individual cell was equivalent to the transfection efficiency observed with a GFP reporter (see Fig. 1c). This allowed also to confirm that viral antigens were detectable only when all the overlapping subgenomic DNA amplicons encompassing the entire viral genome were transfected, ruling out a potential expression before recombination or as a result of unsuccessful recombination events. Using the ISA reverse genetics system previously described in mammalian cells for the flavivirus Japanese Encephalitis virus (JEV, genotype I strain JEV_CNS769_Laos_2009)^18^, we varied the duration of transfection (3 µg of an equimolar mixture of three sub-genomic amplicons) of C6/36 cells at 37 °C from 2 to 48 hours, confirming that 12 hours was the minimum time to obtain a 100% efficacy at 37 °C (data not shown).

The optimised [Lipofectamin 3000 12-hour at 37 °C / 3 µg of an equimolar mixture of sub-genomic amplicons] transfection protocol was subsequently used to rescue a panel of mosquito-borne positive single-stranded RNA arboviruses including a variety of flaviviruses (JEV; Yellow Fever viruses, YFV, strains Asibi and 17D; Zika virus, ZIKV, strains H/PF/2013 and A.taylori-tc/SEN/1984/41662-DAK), the alphavirus Chikungunya virus (CHIKV, strain LR2006 OPY1) and a panel of mosquito (C6/36 and U4.4) and/or mammalian (BHK-21, HEK-293 and SW13) cell lines (Table 1). Cell supernatant media were serially passaged four times using the same cell type to ensure removal of transfected DNA and virus replication was demonstrated using a combination of several criteria (cytopathic effect (CPE), molecular viral load in supernatant at passage 4, genome integrity verification by NGS and production of infectious particles quantified by TCID_50_ determination at passage 2 as previously proposed^18,21^. Results of these experiments are summarized in Table 2 and confirmed the effectiveness of this transfection protocol both in mammalian and insect cells: a CPE was systematically observed from the first passage (except for CHIKV and YFV 17D viruses in C6/36 cells), average amounts of viral RNA detected ranged between 5.4 and 8.6 log_10_ copies/mL and average infectious titers ranged between 4.0 and 8.4 log10 TCID_50_/mL. As expected, NGS analyses confirmed the integrity of the genome and the high genetic similarity (>99.9%) was in accordance with our previous report^24^ with the same JEV strain. Finally, by increasing the number of sub-genomic amplicons of JEV from 3 to 10 DNA fragments with a length ranging between 1 and 4.5 kb, we demonstrated the versatility of this transfection procedure applied in C6/36 mosquito cells as also observed previously with the same virus in mammalian cells with up to six overlapping DNA fragments^18^ (Table 2).

**Table 1:** Summary of the different cell types used to rescue the arboviruses in this study.

**CELLS**	**Viruses**					
	JEV	YFV (Asibi; 17D)	CHIKV	WNV	ZIKV (PF, DAK)	
Mosquito	C6/36	x	x	x	x	x
	U4.4	x				
Mammalian	BHK-21	x	x		x*	x**
	HEK-293	x	x	x*		x**
	SW13	x				

*From Aubry *et al*.^18^ **From Atieh *et al*.^21^.

**Table 2:** Summary of the experiments demonstrating the replication of the recovered viruses.

**Viral strain (Accession Number)**	**Template for subgenomic amplicons**			**Cell line used**	**CPE**	**Amount of viral RNA (Log**_10_ **copies/mL; mean + /− SD)**	**infectious titers(Log**_**10**_ **TCID50/mL; mean + /− SD)**	**Complete genome similarity**
	**Origin**	**Number of fragments**	**DpnI treatment**	**Transfection/ Passage**				
**JEV (KC196115)**	DNS	3	No	U4.4	Yes	6.82+/− 0.20	6.40+/− 0.10	99.9%
	DNS	3	No	C6/36	Yes	7.22+/− 0.21	8.41+/− 0.29	99.9%
	DNS	3	No	BHK-21	Yes	7.09+/− 0.18	7.69+/− 0.12	99.9%
	DNS	3	No	HEK-293	Yes	6,91+/− 0.34	7.98+/− 0.24	99.9%
	DNS	3	No	SW13	Yes	6.65+/− 0.23	7.65+/− 0.24	99.9%
	DNS	4	Yes	C6/36	Yes	7.34+/− 0.09	7.88+/− 0.43	99.9%
	DNS	5	Yes	C6/36	Yes	7.28+/− 0.04	7.46+/− 0.10	99.9%
	DNS	6	Yes	C6/36	Yes	7.33+/− 0.07	7.69+/− 0.12	99.9%
	DNS	8	Yes	C6/36	Yes	6.95+/− 0.14	7.37+/− 0.28	99.9%
	DNS	10	Yes	C6/36	Yes	6.66+/− 0.06	6.49+/− 0.07	99.9%
**ChikV (EU224268)**	I.C.	3	Yes	C6/36	No	7.44+/− 0.09	5.65+/− 0.12	NA
**YFV Asibi (AY640589)**	DNS	3	No	C6/36	Yes	7.18+/− 0.10	7.92+/− 0.2	NA
	DNS	3	No	HEK-293	Yes	7.15+/− 0.08	6.26+/− 0.38	NA
	DNS	3	No	BHK-21	Yes	7.13+/− 0.04	6.34+/− 0.18	NA
**YFV 17D (NC002031)**	DNS	3	No	C6/36	No	5.41+/− 0,26	6.56+/− 0.17	NA
	DNS	3	No	HEK-293	Yes	6.39+/− 0.04	6.22+/− 0.13	NA
	DNS	3	No	BHK-21	Yes	6.24+/− 0.08	6.48+/− 0.05	NA
**Zika PF (KJ776791)**	DNS	3	No	C6/36	Yes	8.38+/− 0.21	4.04+/− 0.14	NA
**Zika DAK (KU955592)**	DNS	3	No	C6/36	Yes	8.58+/− 0.38	5.14+/− 0.07	NA

Description of the origin of the initial material used as template for the production of the overlapping PCR fragments (DNS, *de novo Synthesis*; I.C., infectious clone), the number of transfected amplicons and their treatment by the DpnI restriction enzyme before transfection, the cell lines used for transfection and passages, the presence or absence of cytopathic effect (CPE), the amounts of viral RNA in cell supernatant at the fourth passage by real-time RT-PCR, the infectious titers in cell supernatant at the second passage by TCID_50_ assay and the complete genome similarity assay. For amounts of viral RNA and infectious titers, mean values +/− SD values were represented. *NA: not available.

## Discussion

In conclusion, we describe in the present study a universal ISA-derived method that facilitates the rescue of infectious single-stranded positive-sense RNA viruses in mammalian and mosquito cells. We demonstrated here that temperature during regular lipid-based transfection is a critical parameter even in insect cells that grow at low temperature. Transfection methods using regular lipid-based transfection reagent are supposed to have mainly been optimized to be used on mammalian cell lines that are cultured at 37 °C. Accordingly, when applying the same transfection protocol to other vertebrates or invertebrate cells like fish or mosquito cells, which are cultured at lower temperature (5–15 °C), the efficiency of transfection decreases and is often below 10%^14,25^. While a low percentage of transfection may be sufficient for some studies, the rescue of recombinant viruses from overlapped DNA amplicons often requires higher transfection efficiency. In this respect, we showed here that transfection at 37 °C for 12 hours increases the efficiency and is well tolerated by mosquito cells, allowing to rescue virus using the ISA reverse genetics system. Obviously, this method could therefore be extended to all cells cultured at low temperature provided that an increase of the temperature for few hours is not deleterious for the cells.

The use of the pCMV enables viral RNA to be produced in cells after direct transfection and confers some technical advantages over the use of bacteriophage promoters that require the production of RNA *in vitro*. Based on studies restricted to Drosophila and Spodoptera cell lines^26,27^, regular mammalian promoters such as pCMV have been commonly considered to be weak promoters in insect cells with very low efficacy. However, in the present study pCMV proved to be an effective promoter in mammalian cells as well as in the mosquito cells tested. Thus, pCMV could act as a perfect functional shuttle promoter allowing to rescue viruses into such evolutionary divergent cell origin like *Aedes* mosquito and mammalian cells. However, the use of insect-specific promoters for virus rescue in insect cells and/or in mammalian cells remains to be evaluated and could be in the future an alternative as efficient or even better than pCMV.

We suggest that the ISA procedure combined with the transfection protocol described here could be of interest to those who study biological properties of arboviruses or insect-specific viruses. In particular, such reverse genetics tool could help to better understand the genetic determinants contributing to the host range specificity observed within the *Flavivirus* genus, *i.e*. why some of them are transmitted to vertebrate hosts by arthropod vectors (arboviruses), other infect only insects (insect-specific flaviviruses) and other infect vertebrates without any identified vector (viruses with no known vector). In addition and as previously described^21^, the ISA method facilitates the generation of chimeric viruses because overlapping DNA fragments can be easily exchanged. Using this approach, reverse genetics experiments using such chimeric viruses become possible in mammalian and insect cells. By exchanging genomic regions between flaviviruses with different host range specificity, such experiments may help identifying some genetics determinants of host tropism and vector.

Finally, even if the molecular mechanisms allowing the rescue of viruses from overlapping DNA fragments are currently unknown, this study confirms that these specific mechanisms are present and conserved into evolutionary distant cells, like insect and mammalian cells. The description of these mechanisms is currently in progress and is expected to allow in the future further improvements of reverse genetics for RNA viruses. The description of these mechanisms could also lead to substantial improvements in the rescue efficiency at the cell by cell basis. This could be followed, for example, by immunofluorescent staining for dsRNA since dsRNA is only formed during RNA replication, which at the minimum would require an accurate recombination of the subgenomic amplicons.

## Methods

### Cells

Human adrenal carcinoma SW13 cells were grown at 37 °C with 5% CO2 in RPMI Medium (Life technologies) supplemented with 10% heat-inactivated foetal bovine serum (FBS; Life Technologies) and 1% penicillin/ streptomycin (PS; 5000U mL-1 and 5000µg mL-1; Life Technologies). Human embryonic kidney HEK-293 cells (ATCC number CCL-1573) were grown at 37 °C with 5% CO2 in a minimal essential medium (MEM, Life Technologies) supplemented with 7% FBS, 1% PS and 1% Minimum Essential Medium Non-Essential Amino Acids (MEM NEAA; Life Technologies). Baby hamster kidney BHK-21 cells (ATCC number CCL-10) were grown at 37 °C with 5% CO2 in a MEM medium supplemented with 5% FBS, 5% TPB (Tryptose Phosphate Broth; Life Technologies), 1% L-Glutamine 200 mM (Life Technologies) and 1% PS. Cercopithecus aethiops Vero cells (ATCC number CCL-81) were grown at 37 °C with 5% CO2 in MEM medium supplemented with 5%FBS, 1% L-glutamine, 1% PS. *Aedes albopictus* C6/36 (ATCC number CRL-1660) and U4.4 cells were grown at 28 °C without CO2 in a Leibovitz’s L-15 medium (Life Technologies) with 10% FBS, 5% TPB and 1% PS.

### Viruses

Studies were conducted under biosafety level 3 (BSL3) containment. Viral sequences used in this study correspond to West Nile Virus strain Uganda 1937 (GenBank: M12294), Japanese encephalitis virus (JEV) genotype I strain JEV_CNS769_Laos_2009 (GenBank: KC196115) from Laos, Zika virus (ZIKV) strain H/PF/2013 (GenBank: KJ776791.1, EVAg: 001N-01891) from French Polynesia and strain A.taylori-tc/SEN/1984/41662-DAK (GenBank: KU955592.1, EVAg: 001N-01890) from Dakar, Yellow Fever virus (YFV) strain Asibi (GenBank: AY640589), YFV 17D vaccine strain (GenBank: NC_002031) and Chikungunya virus (CHIKV) strain LR2006 OPY1 (GenBank: DQ443544).

### PCR amplification of cDNA fragments

Amplicons of JEV, CHIKV, YFV (ASIBI, 17D) and ZIKA (PF, DAK) viruses were produced using the Platinum PCR SuperMix High Fidelity kit (Life Technologies). The mixture (final volume, 50 µl) contained 45 µL of SuperMix, 2 µl of DNA template at 1 ng/µL (infectious clone or *de novo* synthesized DNA fragment) and 2 µL of each primer (10 µM working solution were used). Assays were performed on a Biometra TProfessional Standard Gradient thermocycler with the following conditions: 94 °C for 2 min followed by 40 cycles of 94 °C for 15 s, 60 °C for 30 s, 68 °C for 5 min and a final elongation step of 68 °C for 10 min. Size of the PCR products was verified by gel electrophoresis and purified using an Amicon Ultra 0.5 mL Kit (Millipore) according to the manufacturer’s instructions. Primers used are described in the Supplementary Table S2.

### Cell transfection and cell supernatant passages

An equimolar mixture of the amplified DNA fragments was used for transfection. DNA-lipid complex was prepared as followed: 12 µL of Lipofectamine 3000 (Life Technologies) was diluted in 250 µL Opti-MEM medium (Life Technologies) and then mixed with a master solution of DNA which contained 3 µg of DNA and 6 µL of P3000 reagent diluted in 250 µL Opti-MEM medium. After an incubation period of 45 minutes at room temperature, the DNA-lipid complex was added to a 12.5 cm^2^ culture flask of subconfluent cells which contains 1 mL of medium without antibiotics. Each experiment was performed in quadruplicate. After an incubation period of 12 hours at 37 °C with 5% CO_2_, the supernatant medium was removed, cells were washed twice (HBSS; Life Technologies) and 3 mL of fresh medium with antibiotic were added. Cells were then incubated 7 days at standard conditions (28 °C for insect cells, 37% with 5% CO_2_ for mammalian cells).

Cell supernatant media were then serially passaged four times using the same cell types (at day 7 post-transfection and then at day 3–4 post-inoculation): 333 µl of cell supernatant clarified by centrifugation was inoculated into a 12.5 cm^2^ culture flask of confluent cells. After an incubation period of 2 hours, cells were washed twice (HBSS) and 3 mL of fresh medium was added before incubation for 3–5 days.

### GFP reporter assay

The pAc5.1 vector from ThermoFisher Scientific containing the eGFP gene under the control of the *D. melanogaster* actin 5C promoter (kindly gifted by Saw-See Hong) was used. DNA-lipid complex was prepared as described above for virus recovering. After an incubation period of 45 minutes at room temperature, one tenth of the DNA-lipid complex was added per well of a 96 wells plate of subconfluent C6/36 cells which contains 100 µL of medium without antibiotics. Each experiment was performed in quadruplicate. After an incubation period of 12 hours at 28 or 37 °C, the supernatant medium was removed, cells were washed twice (HBSS; Life Technologies) and 100 µL of fresh medium with antibiotic were added. Cells were then incubated 2 days at 28 °C before observed with a Leica DMI8 fluorescence microscope.

### Real-time reverse transcriptase PCR assay

Nucleic acids extraction was performed using 200 µL of clarified cell supernatant medium with the EZ1 mini virus 2.0Kit and the EZ1 Biorobot (both from Qiagen) according to the manufacturer’s instructions. Relative quantification of viral RNA was performed using the GoTaq probe 1-step RT-qPCR system kit (Promega). The mixture (final volume: 20 µl) contained 10 µl of GoTaq probe qPCR Master Mix, 0.5 µL of each primer (10 µM working solution were used), 0.2 µl of probe (10 µM working solution were used), 0.5 µl of Go script RT mix, 0.3 µl of nuclease-free water and 8 µl of extracted nucleic acids. Assays were performed using the CFX96 Touch real-time PCR machine (Bio-Rad) with the following conditions: 50 °C for 15 min, 95 °C for 2 min, followed by 45 cycles of 95 °C for 15 s, 60 °C for 40 s. Data collection occurred during the 60 °C step. The amount of viral RNA was calculated from standard curves using synthetic RNA. Primers used are described in the Supplementary Table S3 or are previously described in^18,21^.

### Tissue Culture Infectious Dose 50 (TCID50) assay

For each determination, a 96-well plate culture of confluent cells was used. C6/36 cells were used for JEV and YFV (Asibi), Vero cells for CHIKV and YFV (17D) and BHK-21 cells for ZIKV (PF and DAK). Wells were inoculated 100 µL of medium and 50 µL of serial 10-fold dilutions of clarified cell supernatant media. The plates were incubated for 7 days and read for absence or presence of CPE in each well. The determination of the TCID_50_/mL was performed using the method of Reed and Muench^28^.

### DpnI enzyme treatment

For chikungunya virus and West Nile virus a bacterial infectious clone was used as template to produce the three overlap fragments. The complete removing of the template was ensured by a digestion step with the restriction enzyme DpnI (New England Biolabs) before transfection. To control the efficiency of this additional step we transfected as a negative control only two cDNA fragments (the first and the second, 3 µg final). This control did not produce any infectious virus.

### Full-length genome sequencing

Verification of the complete genome integrity of JEV was performed at passage 2 as previously described^29^. Remaining reads were mapped using the original sequence of the strain JEV_CNS769_Laos_2009 (GenBank: KC196115) and mutation frequency at each position was calculated (number of reads with a mutation compared to the reference divided by the total number of reads). Primers used for sequencing of the full genome of JEV are detailed in Supplementary Table S4.

## Electronic supplementary material

Supplementary information
