# Supplementary material for: New reverse genetics and transfection methods to rescue arboviruses in mosquito cells
Source: Sci Rep. 2017 Oct 25;7:13983. doi: 10.1038/s41598-017-14522-6 (PMC5656662; doi:10.1038/s41598-017-14522-6)
Supplement: Supplementary file 1 — Supplementary information [file 41598_2017_14522_MOESM1_ESM.pdf]

**New reverse genetics and transfection methods to rescue arboviruses in mosquito cells**

Thérèse Atieh[1], Antoine Nougairède[1], Raphaëlle Klitting[1], Fabien Aubry[1], Anna-Bella Failloux[2], Xavier de Lamballerie[1], and Stéphane Priet[1]

[1]UMR "Émergence des Pathologies Virales" (EPV: Aix-Marseille Univ – IRD 190 – Inserm 1207 – EHESP – IHU Méditerranée Infection), Marseille, France

[2]Arboviruses and Insect Vectors Unit, Department of Virology, Pasteur Institute, Paris, France

# Supplementary information

Supplementary Figure S1: Immunofluorescence staining for viral antigens of West Nile Virus rescued by the ISA method.

Supplementary Table S1: Infectious titers of the rescued viruses in C6/36 cell supernatant at the first passage.

Supplementary Table S2: Primers and probes used for the Real-time RT-qPCR assays.

Supplementary Table S3: Primers used for sequencing of JEV full genome.

Supplementary Table S4: Primers used to obtain cDNA fragments of the different viruses used for transfection.

## Supplemental Figures:

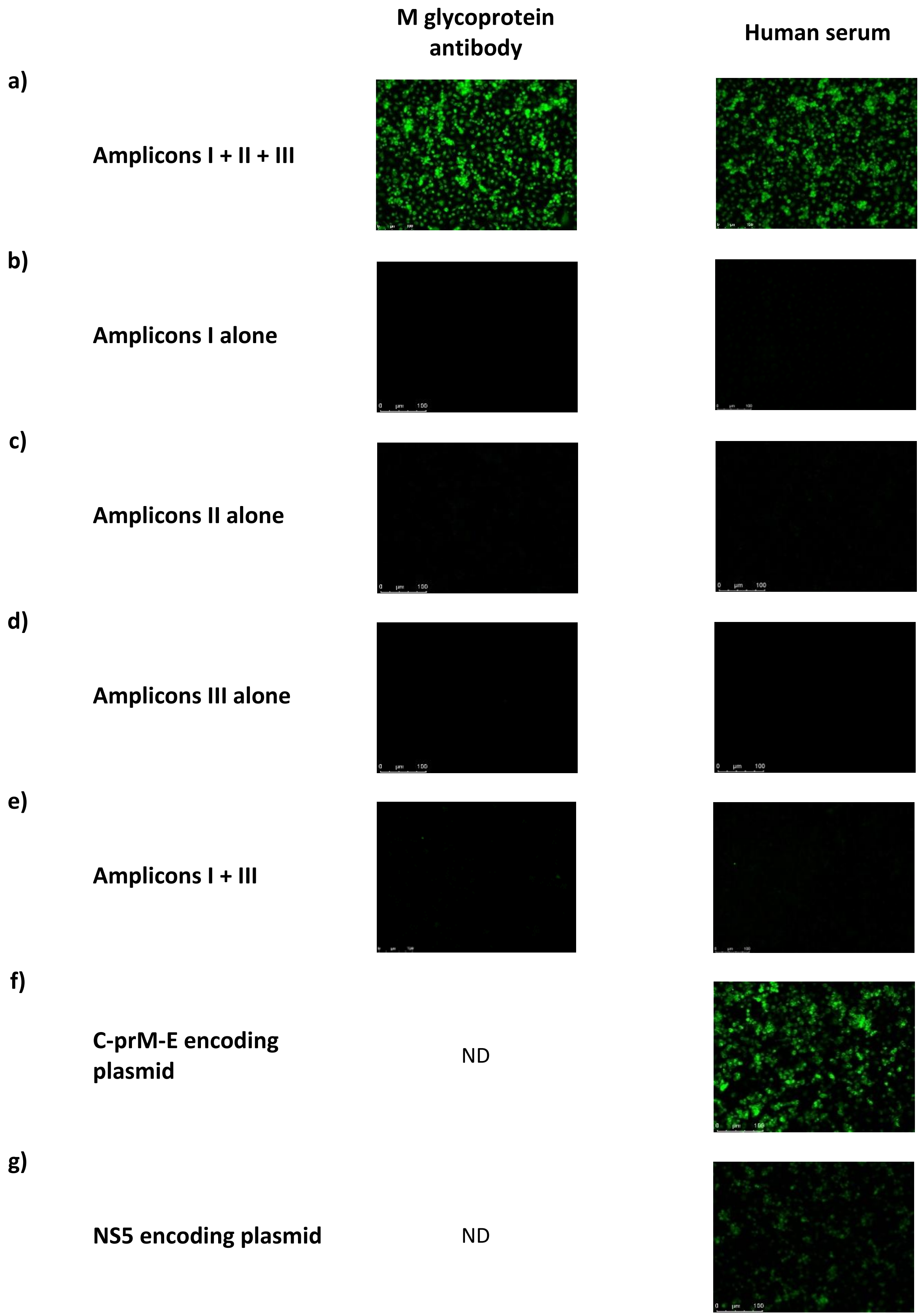

**Supplementary Figure S1: Immunofluorescence staining for viral antigens of West Nile Virus rescued by the ISA method.** The C6/36 cells transfected with all the three overlapping subgenomic DNA amplicons encompassing the entire viral genome of West Nile Virus (fragments I, II and III) (a), the first amplicon alone (b), the second amplicon alone (c), the third amplicon alone (d), the first and third amplicons that do not overlap (e), or a plasmid encoding the C-prM-E polyprotein (f) or the NS5 protein (g) of West-Nile virus under the control of the CMV promoter were probed with a specific M glycoprotein antibody (left panel) or a human serum containing anti-West-Nile virus-specific antibodies (right panel) and a FITC-conjugated secondary antibody. No fluorescence was observed with untransfected cells and with transfected cells stained only with the FITC-conjugated secondary antibody.

## Supplemental Tables:

| A | Temperature of transfection | Samples | Quantity of transfected plasmid (ng) | | | |
|---|---|---|---|---|---|---|
| | | | 2.5 | 5 | 10 | 50 |
| | 37°C | a | 4.95 +/- 0.18 | 4.94 +/- 0.17 | 4.69 +/- 0.20 | 4.96 +/- 0.16 |
| | | b | <1.8¶ | 4.70 +/- 0.18 | 5.49 +/- 0.12 | 4.95 +/- 0.18 |
| | | c | <1.8¶ | 4.69 +/- 0.19 | 5.42 +/- 0.00 | 5.15 +/- 0.11 |
| | | d | 5.15 +/- 0.11 | 5.37 +/- 0.07 | 5.24 +/- 0.11 | 5.19 +/- 0.04 |
| | 28°C | a | <1.8¶ | <1.8¶ | 4.70 +/- 0.20 | 4.95 +/- 0.18 |
| | | b | <1.8¶ | 4.96 +/- 0.16 | 4.94 +/- 0.18 | 4.69 +/- 0.19 |
| | | c | <1.8¶ | <1.8¶ | 4.96 +/- 0.19 | 5.02 +/- 0.28 |
| | | d | <1.8¶ | <1.8¶ | 5.94 +/- 0.16 | 5.24 +/- 0.11 |

| B | Temperature of transfection | Samples | Quantity of transfected DNA(ng) | | |
|---|---|---|---|---|---|
| | | | 100 | 500 | 1000 |
| | 37°C | a | <1.8¶ | <1.8¶ | 6.63 +/- 0.08 |
| | | b | <1.8¶ | <1.8¶ | 6.39 +/- 0.24 |
| | | c | <1.8¶ | <1.8¶ | 6.16 +/- 0.10 |
| | | d | <1.8¶ | <1.8¶ | 7.45 +/- 0.53 |
| | 28°C | a | <1.8¶ | <1.8¶ | 6.14 +/- 0.09 |
| | | b | <1.8¶ | <1.8¶ | <1.8¶ |
| | | c | <1.8¶ | <1.8¶ | <1.8¶ |
| | | d | <1.8¶ | <1.8¶ | <1.8¶ |

¶ Detection threshold of the assay = 1.8 $log_{10}$ $TCID_{50}$/

**Table S1: Infectious titers of the rescued viruses in C6/36 cell supernatant described in Figure 1.**

A: Infectious titers of rescued viruses after transfection of 2.5ng, 5ng, 10ng and 50ng of West Nile Virus infectious clone in C6/36 cells under two different temperature 37°C and 28°C.

B: Infectious titers of rescued viruses by ISA procedure after transfection of 100ng, 500ng and 1000ng of the three DNA overlapping fragments in C6/36 cells at 28°C or 37°C.

Infectious titers are expressed as mean +/- SD of Log10 values. Each experiment was performed in quadruplicate.

| Virus | cDNA fragments | | Primer forward | Position* | Primer reverse | Position* | DpnI enzymatic treatment | Fragment used as negative control |
|---|---|---|---|---|---|---|---|---|
| | number of transfected fragments | length | | | | | | |
| YFV Asibi | 3 | 4818 bp | ¶ CACCCAACTGATCTTCAGCATCT | ¶ | CTATGATAACCACGGTACAAAAGAG | 4012-4037 | No | |
| | | 3041 bp | AGCATCAAATACCATCTTGCCCCT | 3936-3960 | CCGGCCAACTCCAGGGTGAAGC | 6955-6977 | No | |
| | | 4199 bp | GGCAGCCAACGAGCTAGGCAT | 6879-6900 | CTCAGGGTCAATGCCAGCGCTT | £ | No | |
| YFV 17D | 3 | 4800bp | CACCCAACTGATCTTCAGCATCT | ¶ | AACATTCTTTGGATCCTGCACG | 2718-2740 | No | |
| | | 4717 bp | ACTCCCTTGAGCATGAGATGT | 2633-2654 | ACTGTTGGATTCCCATCAACC | 7329-7350 | No | |
| | | 3800 bp | ACTGGTCTCTCATTTTACCTGGA | 7241-7264 | CTCAGGGTCAATGCCAGCGCTT | £ | No | |
| JEV | 3 | 4815 bp | CACCCAACTGATCTTCAGCATCT | ¶ | GAAGAATGATTCTGTAAGTGTCCAG | 4032-4057 | No | |
| | | 3003 bp | CGTTGCCATGCCAATCTTAGCG | 3980-4002 | GGTGCTTGCGTCCTTCCACCAA | 6961-6983 | No | |
| | | 4247 bp | CAAATGAGTATGGAATGCTGGAAAA | 6910-6935 | CTCAGGGTCAATGCCAGCGCTT | £ | No | |
| | 4 | 2394 bp | CACCCAACTGATCTTCAGCATCT | ¶ | CATGGAACCATTCCCTATGGACT | 1613-1636 | Yes | x |
| | | 2519 bp | ACTGGATTGTGAACCAAGGAGTG | 1538-1561 | GAAGAATGATTCTGTAAGTGTCCAG | 4032-4057 | Yes | |
| | | 3003 bp | CGTTGCCATGCCAATCTTAGCG | 3980-4002 | GGTGCTTGCGTCCTTCCACCAA | 6961-6983 | No | x |
| | | 4247 bp | CAAATGAGTATGGAATGCTGGAAAA | 6910-6935 | CTCAGGGTCAATGCCAGCGCTT | £ | No | x |
| | 5 | 2394 bp | CACCCAACTGATCTTCAGCATCT | ¶ | CATGGAACCATTCCCTATGGACT | 1613-1636 | Yes | x |
| | | 2519 bp | ACTGGATTGTGAACCAAGGAGTG | 1538-1561 | GAAGAATGATTCTGTAAGTGTCCAG | 4032-4057 | Yes | |
| | | 1531 bp | CGTTGCCATGCCAATCTTAGCG | 3980-4002 | CAGTTCCACCTTGGTCGCAATG | 5507-5529 | Yes | x |
| | | 1565 bp | ATGTCACCAAACAGGGTGCCCAA | 5418-5441 | GGTGCTTGCGTCCTTCCACCAA | 6961-6983 | Yes | |
| | | 4247 bp | CAAATGAGTATGGAATGCTGGAAAA | 6910-6935 | CTCAGGGTCAATGCCAGCGCTT £ | £ | No | x |
| | 6 | 2394 bp | CACCCAACTGATCTTCAGCATCT | ¶ | CATGGAACCATTCCCTATGGACT | 1613-1636 | Yes | x |
| | | 2519 bp | ACTGGATTGTGAACCAAGGAGTG | 1538-1561 | GAAGAATGATTCTGTAAGTGTCCAG | 4032-4057 | Yes | |
| | | 1531 bp | CGTTGCCATGCCAATCTTAGCG | 3980-4002 | CAGTTCCACCTTGGTCGCAATG | 5507-5529 | Yes | x |
| | | 1565 bp | ATGTCACCAAACAGGGTGCCCAA | 5418-5441 | GGTGCTTGCGTCCTTCCACCAA | 6961-6983 | Yes | |
| | | 2040 bp | CAAATGAGTATGGAATGCTGGAAAA | 6910-6935 | GCGCCGTGCTCCATTGATTCTG | 8928-8950 | Yes | x |
| | | 2336 bp | GGGAGAAACGACCCCGCTTGTG | 8842-8864 | CTCAGGGTCAATGCCAGCGCTT | £ | Yes | |
| | 8 | 1958 bp | CACCCAACTGATCTTCAGCATCT | ¶ | GCATCGAGCTACCGTTGAAATGT | 1177-1200 | Yes | x |
| | | 1270 bp | AGGAGACAGCTGTTTGACAATC | 1055-1077 | GAATGCACCGCCAAATACTTGGT | 2302-2325 | Yes | |

| | | | | | | | |
|---|---|---|---|---|---|---|---|
| | 1876 bp | ACGCTGGGCAAAGCTTTCTCA | 2181-2202 | GAAGAATGATTCTGTAAGTGTCCAG | 4032-4057 | Yes | |
| | 1022 bp | CGTTGCCATGCCAATCTTAGCG | 3980-4002 | GTGGGTAGTCCAAGCTGACTGCT | 4979-5002 | Yes | x |
| | 1193 bp | ACAGATGATGTGCAAGTGATTGTG | 4881-4905 | CAGTGGGCTAAGTTGCTGTCAT | 6052-6074 | Yes | |
| | 1058 bp | AGGTAGAGTCATTCTTGGAAAC | 5924-5946 | GGTGCTTGCGTCCTTCCACCAA | 6961-6983 | Yes | |
| | 2765 bp | CAAATGAGTATGGAATGCTGGAAAA | 6910-6935 | ACTGATCGCCATCCTAGTCACT | 9653-9675 | Yes | x |
| | 1654 bp | CTACGCTCTCAACACATTCACG | 9503-9525 | CTCAGGGTCAATGCCAGCGCTT | £ | Yes | |
| | 750bp | CACCCAACTGATCTTCAGCATCT | ¶ | AAGCCAAGAAGTTCACACAGATAAACT<br>TCTCGGTTCACTAAACGAGCTCTGC | | Yes | x |
| | 1200 bp | AGAAGTTTATCTGTGTGAACTTCT | 0-24 | GCATCGAGCTACCGTTGAAATGT | 1177-1200 | Yes | x |
| | 1270 bp | AGGAGACAGCTGTTTGACAATC | 1055-1077 | GAATGCACCGCCAAATACTTGGT | 2302-2325 | Yes | |
| | 1876 bp | ACGCTGGGCAAAGCTTTCTCA | 2181-2202 | GAAGAATGATTCTGTAAGTGTCCAG | 4032-4057 | Yes | |
| 10 | 1022 bp | CGTTGCCATGCCAATCTTAGCG | 3980-4002 | GTGGGTAGTCCAAGCTGACTGCT | 4979-5002 | Yes | x |
| | 1193 bp | ACAGATGATGTGCAAGTGATTGTG | 4881-4905 | CAGTGGGCTAAGTTGCTGTCAT | 6052-6074 | Yes | |
| | 1058 bp | AGGTAGAGTCATTCTTGGAAAC | 5924-5946 | GGTGCTTGCGTCCTTCCACCAA | 6961-6983 | Yes | |
| | 1038 bp | CAAATGAGTATGGAATGCTGGAAAA | 6910-6935 | CGTAGTAGCTCCAGCCTCCG | 7928-7948 | Yes | x |
| | 1899 bp | GAGGTGGACCGCACTGAAGCA | 7776-7797 | ACTGATCGCCATCCTAGTCACT | 9653-9675 | Yes | |
| | 1654 bp | CTACGCTCTCAACACATTCACG | 9503-9525 | CTCAGGGTCAATGCCAGCGCTT | £ | Yes | |

**Table S2: Primers used to obtain cDNA fragments of the different viruses used for transfection.**

I, II and III designate respectively the first, the second and the third cDNA fragments obtained by PCR. bp: base pair. Primers located respectively at the 5' and 3' terminus of the pCMV (¶) and the HDR/SV40pA (£).

The primers described previously by Aubry *et al* and Atieh *et al* were used to amplified the three overlapped fragments for CHIKV, WNV and ZIKA viruses.

*All positions based on complete viral strain sequence

| Virus | Position | Length | Primer Forward | Probe | Primer Reverse |
|---|---|---|---|---|---|
| **YFV Asibi** | 6474-6579 | 105 | CCCTGATTTCCTGGCTAAAAAA | FAM-TTCTCCACTCTGAGGAAGGCTCT-TAMRA | ATCATTGATAGTGCATTGCGGT |
| **YFV 17D** | 8278-8354 | 76 | TCCACTCATGAAATGTACTACGTGTCT | FAM-AGCCCGCAGCAATGTCACATTTACTGT-TAMRA | GGAGGCGGGATGTTTGGT |

**Table S3: Primers and probes used for the Real-time RT-qPCR assays.**

The primers and probes described previously by Aubry *et al.* and Atieh *et al.* were used to amplified the three overlapped fragments for CHIKV, WNV and ZIKA (H/PF/2013 and A.taylori-tc/SEN/1984/41662-DAK).

| cDNA fragments | | | | | |
|---|---|---|---|---|---|
| **number** | **length** | **Forward** | **position** | **Reverse** | **position** |
| I | 2026 | AGAAGTTTATCTGTGAACTTCT | 1-20 | GGGTCATGTCGTTTAAACTCGC | 2004-2026 |
| II | 2137 | GCGGACACAGGCCATGGAA | 1920-1939 | GAAGAATGATTCTGTAAGTGTCCAG | 4032-4057 |
| III | 3003 | CGTTGCCATGCCAATCTTAGCG | 3980-4002 | GGTGCTTGCGTCCTTCCACCAA | 6961-6983 |
| IV | 2207 | CAAATGAGTATGGAATGCTGGAAAA | 6910-6935 | CCACATGAACCAAATAGCCCTAC | 9094-9117 |
| V | 1993 | CAATGGAGCACGGCGCGGG | 8934-8953 | GGCGCTCTGTGCCTAGTAGC | 10907-10927 |

**Table S4. Primers used for sequencing of JEV full genome.**

Sequencing of JEV full genome was performed by dividing the genome into five overlapped fragments. This table showed the set of specific primers used during RT-PCR assays to generate the overlapped fragments.

## Supplemental Methods:

### Immuno-fluorescence assay

Immunofluorescence staining assay was performed on C6/36 cells transfected for 12 hours at 37°C with the subgenomic amplicons used to rescue the West Nile virus through the ISA method [1]. In this model, three overlapping subgenomic DNA amplicons encompassing the entire viral genome (fragments I, II and III) are used to recover an infectious virus. One, two or the three subgenomic amplicons of the West Nile virus model were transfected in C6/36 cells. One day post-transfection cells were seeded on slide and incubated for 24h at 28°C. After then, cells were washed gently three times with PBS, and fixed in 4% paraformaldehyde for 20 min at room temperature. After three washes with PBS, cells were permeabilized by 0.2% Triton X-100 for 5 min at room temperature. Cells were washed once with PBS, and then incubated 1 hour in blocking solution containing 0.5% bovine serum albumin (BSA). Then, cells were probed overnight at 4°C with a specific M glycoprotein antibody (Abcam) diluted to 1/50 in PBS-1% BSA or a human serum containing anti-West-Nile virus-specific antibodies [2] diluted to 1/100 in PBS-1% BSA. After three washes with blocking solution, cells were incubated for 1hour at room temperature with the second antibody an anti-rabbit or an anti-human diluted at 1/100 in PBS-1% BSA. Both secondary antibodies are conjugated with fluorescein isothiocyanate (FITC). Slides were washed three times with PBS, and then visualized with a DMI8 Leica microscope and an FITC excitation and emission filter set. As controls for the presence of specific antibodies in our human immune serum, C6/36 cells were transfected at 37°C with in-house plasmids encoding the C-prM-E polyprotein or the NS5 protein of West-Nile virus under the control of the CMV promoter.

**Supplemental References:**


1 Aubry, F. *et al.* Single-stranded positive-sense RNA viruses generated in days using infectious subgenomic amplicons. *J Gen Virol* **95**, 2462-2467, doi:10.1099/vir.0.068023-0 (2014).

2 Gake, B. *et al.* Low seroprevalence of Zika virus in Cameroonian blood donors. *Braz J Infect Dis* **21**, 481-483, doi:10.1016/j.bjid.2017.03.018 (2017).
